# Comparison of severity of illness scoring systems in the prediction of hospital mortality in severe sepsis and septic shock

**DOI:** 10.4103/0974-2700.70761

**Published:** 2010

**Authors:** Colleen A Crowe, Erik B Kulstad, Chintan D Mistry, Christine E Kulstad

**Affiliations:** Department of Emergency Medicine, Advocate Christ Medical Center, Oak Lawn, IL, USA

**Keywords:** Severity of illness index, sepsis, mortality, septic shock, critical care

## Abstract

**Background:**

New scoring systems, including the Rapid Emergency Medicine Score (REMS), the Mortality in Emergency Department Sepsis (MEDS) score, and the confusion, urea nitrogen, respiratory rate, blood pressure, 65 years and older (CURB-65) score, have been developed for emergency department (ED) use in various patient populations. Increasing use of early goal directed therapy (EGDT) for the emergent treatment of sepsis introduces a growing population of patients in which the accuracy of these scoring systems has not been widely examined.

**Objectives:**

To evaluate the ability of the REMS, MEDS score, and CURB-65 score to predict mortality in septic patients treated with modified EGDT.

**Materials and Methods:**

Secondary analysis of data from prospectively identified patients treated with modified EGDT in a large tertiary care suburban community hospital with over 85,000 ED visits annually and 700 inpatient beds, from May 2007 through May 2008. We included all patients with severe sepsis or septic shock, who were treated with our modified EGDT protocol. Our major outcome was in-hospital mortality. The performance of the scores was compared by area under the ROC curves (AUCs).

**Results:**

A total of 216 patients with severe sepsis or septic shock were treated with modified EGDT during the study period. Overall mortality was 32.9%. Calculated AUCs were 0.74 [95% confidence interval (CI): 0.67–0.81] for the MEDS score, 0.62 (95% CI: 0.54–0.69) for the REMS, and 0.59 (95% CI: 0.51–0.67) for the CURB-65 score.

**Conclusion:**

We found that all three ED-based systems for scoring severity of illness had low to moderate predictive capability. The MEDS score demonstrated the largest AUC of the studied scoring systems for the outcome of mortality, although the CIs on point estimates of the AUC of the REMS and CURB-65 scores all overlap.

## INTRODUCTION

Sepsis remains a leading cause of death in critically ill patients in the emergency department (ED).[1] Rapid and intensive treatment of septic patients with early goal directed therapy (EGDT) has been shown to decrease mortality.[2] Other treatments, such as with drotrecogin alfa, are based on measures of patients’ severity of illness.[3] Because of the need for rapid and consistent stratification of patients’ illness severity to treat individual patients and to reliably compare patient cohorts across geographic boundaries, a number of scoring systems have been developed that circumvent the complexity and time constraints of traditional scoring systems developed for use in the Intensive Care Unit setting.[4–7] These newer scores include the mortality in emergency department sepsis (MEDS) score, the rapid emergency medicine score (REMS), and the confusion, urea nitrogen, RR, blood pressure, 65 years and older (CURB-65) score.[8–10]

The MEDS, REMS, and CURB-65 scoring systems have been applied to various subsets of patients, with variable results. The 28-day mortality predictions of all three scores were evaluated in admitted ED patients with suspected infection and found to be useful.[11] The predictive capability of the MEDS score for 28-day mortality in patients with severe sepsis or septic shock was found to be superior to that of the Acute Physiology and Chronic Health Evaluation (APACHE) II score.[12] The application of these newer scores to the increasing number of patients treated with EGDT, however, has been limited, with one analysis suggesting poor accuracy for in-hospital mortality in this patient population.[13]

In order to further analyze the performance of these newer scoring systems specifically in patients with sepsis severe enough to require treatment with EGDT, we sought to compare the accuracy of the MEDS, REMS, and CURB-65 scoring systems in predicting in-hospital mortality. We restricted our analysis to ED patients treated with our hospital’s modified EGDT protocol.

## MATERIALS AND METHODS

### Study design

This was a secondary analysis of data from a study that prospectively identified patients treated with modified EGDT from May 2007 through May 2008.[14] The study was approved by the hospital’s Institutional Review Board, with a waiver of informed consent.

### Study setting and population

This study was conducted at a large tertiary care suburban community hospital with over 85,000 ED visits annually and nearly 700 inpatient beds.

### Study protocol

We analyzed data from all patients who had been enrolled in our ongoing prospective study of the outcomes of septic patients treated with our modified EGDT protocol. Specifically, we included all patients with two or more systemic inflammatory response syndrome (SIRS) criteria and a suspected or documented infection. SIRS criteria included: (a) temperature > 38°C or < 36°C; (b) heart rate > 90 beats/minute; (c) respiratory rate (RR) > 20 breaths/minute or PaCO2 < 32 mmHg; (d) WBC > 12,000/μL or < 4000/μL, or differential cell count with >10% bands. Patients who met two or more SIRS criteria and had suspected or documented infection were further categorized as having (a) septic shock if mean arterial pressure (MAP) was <65 or systolic blood pressure (SBP) was <90 after appropriate intravenous fluids (IVF) bolus (20.30 mL/kg over 30 min) or (b) severe sepsis if lactate was ≥ 4 mmol/L or with an evidence of ≥1 organ dysfunction.[15] We excluded patients with age less than 18, Do Not Resuscitate (DNR) status, pregnancy, or evidence of traumatic injury.

We asked all attending and resident physicians to alert one of the study coordinators to any patient meeting study criteria and being treated with EGDT. To ensure that all eligible patients were identified, we also utilized daily monitoring of our electronic tracking board by a research assistant, who identified patients both by ED diagnosis (including the terms Sepsis, Shock, Pneumonia) and by documented procedures [including placement of central line and central venous pressure (CVP) monitoring].

EGDT elements include the initiation of broad-spectrum antibiotics within 4 hours, central line placement with CVP monitoring, use of supplemental oxygen or mechanical ventilation as deemed necessary, and achievement of hemodynamic goals within 6 hours.[2] The EGDT protocol used at our institution incorporates all of the elements initially proposed by Rivers *et al*., with the exception of continuous central venous oxygen saturation monitoring using a specialized proprietary catheter.[2] As a substitute for this continuous monitoring, our protocol uses a periodic sampling of central venous blood for oxygen saturation, and consequently is referred to as “modified” EGDT.

### Methods of measurement

A standardized abstraction form was created for data collection prior to the enrollment of patients. Initial recorded vital signs obtained in the ED were used. Chart abstractors met at the start of the study to define methods and were experienced in the methods of chart abstraction, but were unable to be blinded to study purpose. Any data point not recorded in the medical record was abstracted as normal or not present, in accordance with methodology utilized in the original derivation of these scoring systems.[8,9] Abstraction of various data points was dependent on the availability of investigators and occurred at any point in the patient’s treatment course, including after admission to the hospital, and in some cases, after discharge. Periodic meetings were held throughout the progress of our study, but we did not attempt to test formal inter-rater agreement.

The MEDS score incorporates nine variables, assigned 2–6 points, with the maximum score being 27.[8] The variables are age, nursing home residence, lower respiratory tract infection, bandemia, thrombocytopenia, tachypnea or hypoxemia, shock, altered mental status, and terminal illness [Table 1]. Specific definitions for these variables were not modified from the original description.[8]

**Table 1:** Variables used in the calculation of the MEDS score (the MEDS score ranges from 0 to 27)

Variable	Points	Comment
Terminal illness	6	Rapidly fatal illness such as metastatic cancer with perceived 30-day mortality
Age >65 years	3	
Tachypnea or hypoxia	3	RR > 20 breaths/min, requiring O_2_ by mask, O_2_ saturation < 90%
Shock	3	SBP < 90 after appropriate IVF bolus
Thrombocytopenia	3	<150,000 cells/mm^3^
Bandemia	3	>5%
Nursing home resident	2	
Lower respiratory tract infection	2	
Altered mental status	2	By history or examination

The REMS is divided into six variables, with the maximum score being 26.[16] Five of the variables, the Glasgow coma scale (GCS), RR, peripheral oxygen saturation, MAP, and pulse rate, contribute up to 4 points each, while age contributes a maximum of 6 points. We used the modified REMS (mREMS), attributing any change in mental status (described in either the initial history or the physical examination section of the chart) a score of 1, because many of our patients lacked a recorded initial GCS [Table 2].[11]

**Table 2:** Variables used in the calculation of the REMS and the mREMS which replaces the GCS value with a binary presence or absence of confusion (the REMS ranges from 0 to 26, mREMS from 0 to 23)

	0	1	2	3	4	5	6
Age (years)	<45		45–54	55–64		66–74	>74
Heart rate (beats/min)	70–109		110–139 or 40–54	140–179 or 40–54	>179 or <39		
RR (breaths/min)	12–24	25–34 or 10–11	6–9	35–49	>49 or <5		
MAP (mmHg)	70–109		110–129 or 50–69	130–159	>159 or <49		
Peripheral O_2_ saturation (%)	>90	86–89		75–85	>75		
GCS	>13	11–13	8–10	5–7	<5		
Modified altered mental status (AMS) (yes/no)?	No	Yes					

The CURB-65 score depends on five variables: confusion, blood urea nitrogen (BUN), RR, hypotension, and age.[10] Each variable contributes one point to the total score, the maximum score thus being 5 [Table 3]. Specific definitions for these variables were not modified from the original description.[10]

**Table 3:** Variables used in the calculation of the CURB-65 score (the CURB-65 score ranges from 0 to 5)

Variable	Points	Comment
Confusion	1	
Urea > 7 mmol/L	1	>19.6 mg/dL
RR > 30 breaths/min	1	
Hypotension	1	SBP < 90 or DBP < 60 mmHg
Age ≥ 65	1	

### Outcome measures

Our primary outcome was in-hospital mortality.

### Data analysis

Baseline demographic and clinical characteristics are described using means with 95% confidence intervals (CIs) for normally distributed data and medians with interquartile ranges (IQRs) for non-normal data. We measured the mortality rate and calculated 95% CIs around the point estimate. Area under the receiver operating characteristic (ROC) curve (AUC) was used to quantify the performance of the scoring systems. Analyses were performed using SPSS version 15.0 (SPSS Inc., Chicago, IL, USA).

## RESULTS

### Characteristics of study subjects

A total of 216 patients were treated with our modified EGDT protocol during the 13-month study period. Their age ranged from 22 to 97 years, with a median age of 71.5 (IQR of 59–81) years; 50.2% of the patients were males. Baseline characteristics of survivors and non-survivors are shown in Table 4. Specific etiologies of sepsis identified in our patient population, and the numbers of patients with each diagnosis, are as follows: pneumonia (81 patients), urosepsis (49 patients), multiple etiologies (38 patients), gastrointestinal (16 patients), bacteremia (15 patients), wound (4 patients), and unidentified (13 patients). Other authors have also noted no causative organisms in a sizeable number of patients with the clinical presentation of sepsis.[17]

**Table 4:** Distribution of patient characteristics among survivors and non-survivors

	Baseline characteristics		*P*
	Survivors	Non-survivors	
Age (years)	69 (IQR 58–78)	77 (IQR 60–86)	0.01
Gender (% male)	51%	49.3%	0.81
Heart rate (beats/min)	114 (95% CI: 109–119)	114 (95% CI: 107–120)	0.95
RR (breaths/min)	24 (95% CI: 23–26)	25 (95% CI: 22–27)	0.56
MAP (mmHg)	55 (95% CI: 52–58)	52 (95% CI: 48–57)	0.37
Peripheral O_2_ saturation (%)	94 (95% CI: 92–95)	93 (95% CI: 91–95)	0.49
Altered mental status (% = yes)	61%	77.5%	0.01
Nursing home resident	62.1%	54.9%	0.32
Lower respiratory infection	40.7%	49.3%	0.23
Age > 65 years	57.2%	66.2%	0.20
Bands > 5%	40%	57.7%	0.14
Platelets < 150,000/mm3	17.9%	32.4%	0.02
Shock	88.3%	94.4%	0.08
Tachypnea or hypoxia	69.7%	88.7%	0.002
Terminal illness	4.8%	32.4%	< 0.001
Urea > 7 mmol/L	85.5%	87.3%	0.72
RR >30 breaths/min	19.3%	22.5%	0.58
Hypotension	19.3%	22.5%	0.29

### Outcomes

Among the 216 patients included in this study, 145 were ultimately discharged alive, for an in-hospital mortality of 32.9% (95% CI: 26.6–39.2). The hospital length of stay (LOS) ranged from 1 to 45 days, with a median of 8 days and an IQR of 4.3–13 days. One hundred eighty-three patients had septic shock (85%; 95% CI: 80–90%), while 24 patients met treatment criteria by the presence of end-organ dysfunction (11%; 95% CI: 8–16%), and 9 met criteria with a lactate greater than 4 (4%; 95% CI: 2–8%).

**Figure 1:**
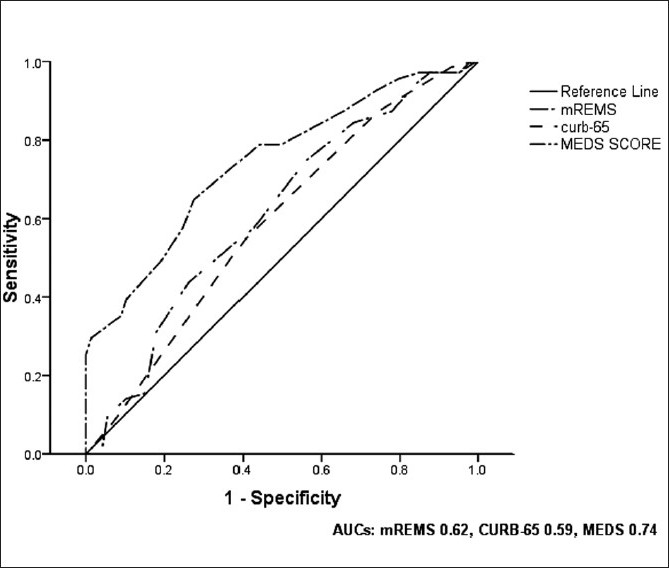
ROC curves

**Figure 2:**
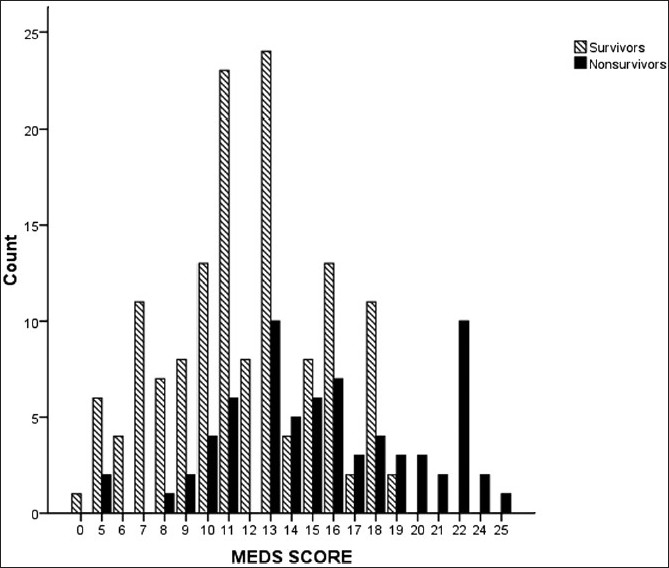
MEDS score and survival rates

The median MEDS score was 13 (IQR 10–16). ROC curve analysis yielded an AUC of 0.74 (95% CI: 0.67–0.81) [Figure 1]. Survivors had a median MEDS score of 11 (IQR 9–14.5) compared to non-survivors, whose median MEDS score was 15 (IQR 13–20) [Figure 2].

**Figure 3:**
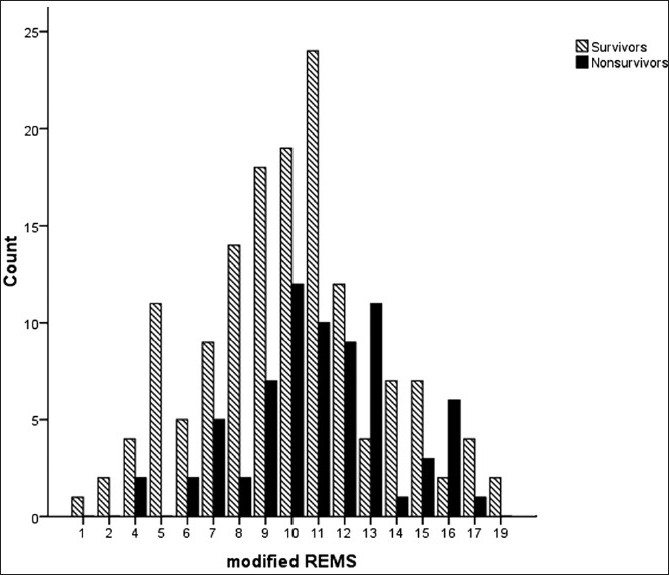
mREMS and survival rates

The median mREMS was 10 (IQR 8–12). ROC curve analysis yielded an AUC of 0.62 (95% CI: 0.54–0.69) [Figure 1]. Survivors had a median mREMS of 10 (IQR 8–12) compared to non-survivors with a median mREMS of 11 (IQR 9–13) [Figure 3].

**Figure 4:**
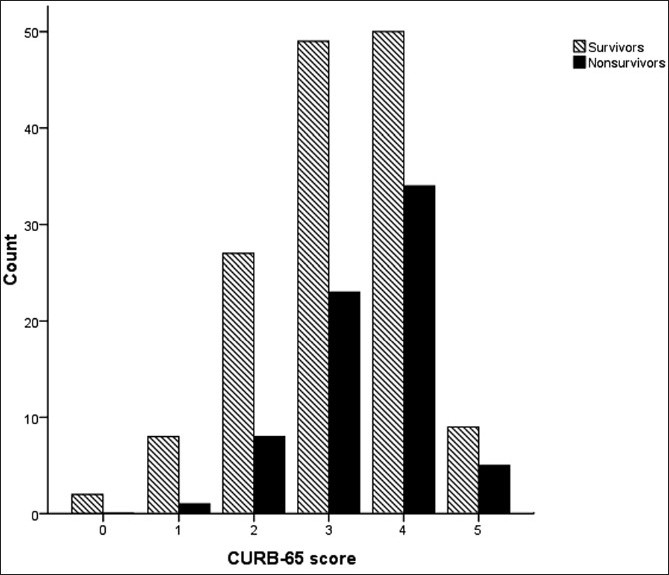
CURB-65 score and survival rates

The median CURB-65 score was 3 (IQR 3–4). ROC curve analysis yielded an AUC of 0.59 (95% CI: 0.51–0.67) [Figure 1]. Survivors had a median CURB-65 score of 3 (IQR 2–4) compared to non-survivors, whose median CURB-65 score was 4 (IQR 3–4) [Figure 4].

### Sensitivity analysis

Many of our patients arriving via ambulance were already receiving supplemental oxygen; consequently, no peripheral pulse oximetry without supplemental oxygenation was recorded for these patients, which probably led to a false lowering of their REMS. We analyzed the effect of this factor by changing the points assigned for pulse oximetry. If the patient’s score was 0 or 1 and the patient was noted to be hypoxic or tachypneic by the MEDS score, they were assigned a value of 2 (the midpoint of the REMS range). This changed the AUC only slightly, from 0.63 (95% CI: 0.55–0.70) to 0.65 (95% CI: 0.57–0.72). Likewise, because of subjectivity inherent in two of the variables of the MEDS score (a change in mental status and the presence of a terminal illness), we reanalyzed our data after omitting these two variables from the calculation of the MEDS score. This resulted in a decrease in the AUC from 0.74 to an AUC of 0.67.

## DISCUSSION

Sepsis remains a leading cause of death in the ED, with mortality estimates between 25 and 50%. As novel treatments such as nitric oxide and recombinant proteins are evaluated, comparisons of their effectiveness are dependent on easily obtainable measures of severity of illness of the population in which they are applied.[18,19] Implementation of new treatments is difficult to target those patients who would benefit the most.

The CURB-65 score, which was originally designed for studies of patients with pneumonia, incorporates the least number of variables, and except for “presence of confusion,” these variables are easily obtained.[10] Confusion can be hard to determine in patients with some degree of baseline dementia or from chart review, but because this variable contributes 20% of the maximum score, it has the potential to skew the score significantly. Perhaps because CURB-65 includes the fewest variables, we found this scoring system to be the least accurate in predicting mortality. The use of CURB-65 as a predictor of mortality has been validated in patients with pneumonia, and in patients with any suspected infection, but some have found its predictive value in patients with pneumonia to be poor.[11,20,21] Studies of patients with the lowest mortality rates (ranging from 6.7 to 9.4%) found high AUCs of 0.80–0.87.[20,22,23] Although a study with a mortality rate of 12% demonstrated an AUC of 0.84, the study with the highest mortality (15.4%) also had the lowest AUC of 0.69, suggesting a dependence of score performance on the population examined.[21,24]

The REMS was developed as an abbreviated version of the APACHE II score for use in all admitted non-surgical ED patients.[9,25] The original calculation of the REMS requires a patient’s GCS to be determined, which is not commonly done with septic patients. Howell *et al*. described an mREMS that had a similar predictive capability, replacing the scale of the GCS with a binary presence or absence of confusion.[11] We used this modification in our calculations. The complexity of this scoring system, with each variable studied being assigned a different value depending on how much it is above or below normal, made its use more difficult than the other two scores.

Earlier studies indicated a good correlation between the REMS and mortality in all non-surgical admitted patients, with AUCs of 0.85–0.91 and mortality rates of 2.4–11.3%.[9,16,25] When only admitted patients with suspected infection were studied, the AUC remained impressive at 0.80 (mortality rate of 3.9%).[11] When Goodacre *et al*. studied admitted ED patients who arrived by ambulance, the mortality rate was higher (12.7%) and the AUC of 0.74 was not as robust.[26]

The MEDS score was developed by Shapiro *et al*., as a way to determine which of the patients with suspected infection are at high risk for death.[8] This score incorporates nine variables as compared with only five in the CURB-65 score. The laboratory values and physiologic data for the MEDS score are easy to obtain in the ED, but two of the variables, a change in mental status and the presence of a terminal illness, can be subjective and difficult to determine from the medical record. We examined the performance of this score without these variables, but found a lower predictive performance, with an AUC of 0.67.

The initial set of patients used for the MEDS score’s derivation and validation were the admitted patients who had a blood culture ordered in the ED, and demonstrated an AUC of 0.78–0.82.[8] Howell *et al*. studied the patients admitted from the ED with suspected infection, and found the AUC to be good at 0.85.[11] As the patient population studied changed to ED patients with SIRS, the score continued to perform well, with an AUC of 0.88.[27] In more severely ill patients, however, the predictive capability started to decline. Chen *et al*. studied ED patients with severe sepsis, and found an AUC of 0.75.[12] The most recent studies, focusing on patients with severe sepsis and septic shock, show AUCs of 0.60 and 0.61, respectively.[13,28] Our results with the MEDS score, obtained from patients with severe sepsis and septic shock, show an AUC of 0.74, but this value was not as high as that found in studies of less severely ill patients.

### Limitations

This study is limited by the collection of data at a single center on a single sample of patients, without additional assessments on multiple populations. Additionally, although our patients were prospectively identified, various components of the scoring systems were obtained by chart abstraction. Certain variables may have been incorrectly categorized if they were not recorded or were incorrectly recorded by the physician or nurse completing the chart.

The mREMS was calculated on the basis of the initially recorded vital signs in our study. For peripheral oxygen saturation, however, a value was often not recorded on very ill patients while they were breathing only room air. This may have decreased the predictive capability in our patient population.

Unmeasured variables such as clinical appearance may also contribute bias to this study. Because the initiation of EGDT is at the discretion of the treating physicians, additional factors may influence the decision to implement the treatment protocol. It is possible that patients who appeared less ill, but may have met strict inclusion criteria, may not have received EGDT and therefore would not have been included in our study.

## CONCLUSION

We found that all three ED-based systems for scoring severity of illness had low to moderate predictive capability. The MEDS score demonstrated the largest AUC of the studied scoring systems for the outcome of mortality, although the CIs on point estimates of the AUC of the REMS and CURB-65 scores all overlap. Patient care should not be altered based on values derived from these scoring systems.
